# Autonomy in learning: Predictability modulates the beneficial effect of choice on memory

**DOI:** 10.3758/s13421-026-01863-9

**Published:** 2026-02-25

**Authors:** Zhaoqi Zhang, Lieke L.F. van Lieshout, Harold Bekkering

**Affiliations:** Donders Institute for Brain, Cognition and Behaviour, Radboud University, Nijmegen, The Netherlands

**Keywords:** Autonomy, Choices, Prediction, Learning motivation, Memory

## Abstract

When people are offered the opportunity to choose, they tend to learn better. However, the cognitive mechanisms of the beneficial effect of choice on memory remain to be further investigated. On the one hand, self-determination theory has proposed that choices themselves might be motivating in and of themselves, even when their consequences are not predictable. On the other hand, from a predictive coding perspective, choices facilitate learning because they enhance the prediction of upcoming information. To delve deeper into these two interpretations, we conducted two experiments using a learning task in which we independently manipulated choice and predictability. In Experiment 1, we included 55 participants, while Experiment 2 included 56 participants. In half of the blocks, participants could choose which object they wanted to see (choice), while in other blocks, the choices were made for them (no choice). Meanwhile, in half of the choice and no-choice blocks, they would always see the selected object (high-predictability), while in other blocks, they had a 50% chance to see the selected object (low-predictability). The memory accuracy was tested by a recognition task. In both experiments, we found that the facilitatory effect of choice on memory accuracy existed under both high and low predictability. However, this effect was smaller under low predictability. These findings provided evidence for both interpretations: choices enhance learning because they are motivating by themselves, meanwhile facilitating prediction processes. This study has important implications for education, urging teachers to offer choices to students and to satisfy students’ needs in classroom settings.

**Supplementary Information:**

The online version contains supplementary material available at 10.3758/s13421-026-01863-9.

## Introduction

The world around us offers an enormous number of possibilities to satisfy our needs. For example, when we learn to play the piano, we must decide which music style to start with. The fact that you can choose might make you happy in and of itself: simply having the opportunity to choose which style to play might delight you. However, choices also lead to different consequences. Thus, perhaps the choice itself is not motivating, but the fact that you can predict the outcome of the choice (the music) is. The current study aims to investigate how choices affect our learning under different predictabilities.

Learning and memory formation can be promoted when people can make their own choices. According to the self-determination theory (SDT), autonomy (i.e., the freedom to choose), is defined as a key motivational drive for learning, often referred to as intrinsic motivation. People are more likely to continue to learn when their need for autonomy is satisfied (Ryan & Deci, 2020). Indeed, a multitude of studies have demonstrated that the freedom to choose boosts memory encoding (e.g., Ding et al., 2021; Lima et al., 2023; Murty et al., 2015; Rotem-Turchinski et al., 2019). For example, Murty et al. (2015) found higher memory accuracy in a condition in which participants could choose which picture they would like to see (choice condition), compared with a condition in which the choice was made by the computer (no-choice condition). Similar beneficial effects of choice on learning performance have been found when choice was manipulated as having volitional control over the spatial learning trajectory over a map of objects (van Lieshout et al., 2023; Voss et al., 2011), or as choosing the time of viewing object pictures (Markant et al., 2014). Therefore, the beneficial effect of active choices on learning is consistent and stable. Nevertheless, little is known about its cognitive mechanisms.

Multiple cognitive processes could drive these choice-related memory benefits. First, it might be the case that having a choice is motivating in and of itself. According to Ryan and Deci (2020), choices are internally satisfying and motivate people to continue to learn. For example, DuBrow et al. (2019) found that people naturally preferred symbols associated with the choice condition compared with the no-choice condition. These symbols were not linked with the pictures that they were asked to remember. Also, a positive correlation was found between the magnitude of the choice-induced preference over the symbols and the memory accuracy of the pictures shown after the selection of the symbols across participants. These findings suggested that inconsequential choices could enhance memory formation (Ding et al., 2021; Murty et al., 2015; Rotem-Turchinski et al., 2019), likely by inducing a more pleasant emotional response (DuBrow et al., 2019). Taken together, having the opportunity to choose is fulfilling in and of itself and can enhance learning, regardless of the choice outcomes.

However, we build up a causal relationship between a voluntary action and the appearance of a certain outcome following that action (Desantis et al., 2011; Moore & Haggard, 2008). Therefore, another line of reasoning suggests that the beneficial effects of choice on learning are due to enhanced predictability of the choice outcomes. According to active inference under the free-energy principle (Friston et al., 2013), active choices help to predict what you are going to perceive (i.e., choices help you to build a prior belief of the upcoming perceptual information). More specifically, when people make choices, they predict that the outcome will be in line with their choice. These consequential choices reduce the prediction error over time (Peterson et al., 2011). On the contrary, if the choices do not predict the outcome, the prediction error cannot be reduced, and learning is not enhanced. Hence, only choices that are associated with smaller prediction errors will improve learning (Peterson et al., 2011). For instance, some studies have suggested that the beneficial effects of choice on learning disappear when the choice is not predictive of the outcome (Chambon et al., 2020; Katzman & Hartley, 2020; Schneider et al., 2018).

By means of two experiments, we aimed to investigate the cognitive mechanisms of the beneficial effects of choice on learning. Specifically, we aimed to unravel whether the beneficial effect of choice on learning stems from the inherent motivation from choice itself (i.e., independent from the choice outcome) or if it relies on the correct prediction of the outcome of the choice (i.e., dependent on the choice outcome). To address this inquiry, we designed a learning experiment in which we independently manipulated two factors: (1) the presence of a choice itself (yes or no), and (2) the predictability of the choice outcome (high or low). In each trial, participants were presented with two object names. These names corresponded to actual object images participants had to remember. Participants could sometimes choose which of these two objects they wanted to learn (choice condition), and sometimes the choice was made for them (no-choice condition). After the selection, participants saw one of the object images. In high-predictability blocks, this object image always corresponded to the object name that they chose or that was chosen for them. In other words: participants could predict which of the object images they would see. In low-predictability blocks, participants could not accurately predict which of the two object images would be revealed to them. This was the case because participants would see the selected object in half of the trials but would see the not selected object in the other half of the trials. Afterwards, participants’ learning performance was evaluated by a separate recognition memory test.

Given the two explanations regarding the source of the choice effect outlined above, we raise two possible hypotheses. If the choice effect on learning comes purely from the inherent motivation caused by making a choice, the beneficial effect of choice on memory accuracy would be present in both high- and low-predictability scenarios (e.g., Murty et al., 2015). Alternatively, if the positive effect of choice on learning solely comes from the effective prediction of choice outcomes, the beneficial effect of choice on memory accuracy would be present in high-predictability scenarios, but not when participants could not predict the upcoming picture (e.g., Katzman & Hartley, 2020). These experiments will help us to further understand the beneficial effects of choice on learning performance and to better utilize choices in industrial and educational contexts.

## Experiment 1

### Methods

#### Preregistration and data and code availability

Experiment 1 and its analyses were preregistered on the Open Science Framework (10.17605/osf.io/tnqre). All data and code used for stimulus presentation and analyses of both experiments are freely available on the Donders Repository (10.34973/rn1x-d471).

#### Participants

To determine the sample size of our experiments, we conducted a power analysis with MorePower (Campbell & Thompson, 2012). The power analysis suggested that we need at least 56 participants to detect a large effect size (partial eta^2^ = 0.14) with 80% power for the interaction between choice and predictability conditions using a 2 × 2 repeated-measures ANOVA.

We recruited a total of 58 participants, of which 55 (age = 22.7 ± 3.7 (*M* ± *SD*) years; 37 females, 17 males, one non-binary) were included for the final analysis. One participant (57 years old) was excluded because his age was more than 3 standard deviations removed from the average age of the participant sample. Another participant was excluded because of a procedural mistake by the experimenter, and one participant was excluded for unavoidable disruption during the learning task. Participants all reported their English fluency as “very good” and had normal or corrected-to-normal vision.

The experiment was approved by the Ethics Committee of the Faculty of Social Sciences (ECSW) at Radboud University, Nijmegen, the Netherlands, under the general ethics approval for standard studies conducted at the Donders Centre for Cognition (ECSW.2018.115). Prior to participation, all participants gave written informed consent according to the Declaration of Helsinki and confirmed that they were all over 18 years old.

#### Procedure

To test the effect of choice on memory accuracy under different predictabilities, we designed a task consisting of a learning phase and a memory phase. The experiment was performed using Presentation® software Version 23.0 (Neurobehavioral Systems, Inc., 2023).

For the learning phase of the experiment, 448 pictures of unique objects were selected from a database for visual stimuli (Brady et al., 2008; Konkle et al., 2010). Before object presentation, the names of the objects were presented on the screen. These object names were taken from the original database. All the names were checked and, when necessary, corrected by two native English speakers. For each participant, the 448 objects were randomly combined into 224 pairs. During the learning phase, only one object of each pair was made visible to the participants, and the participants were instructed to remember these 224 objects as well as possible. During the memory phase, participants were tested on 448 objects, consisting of the 224 objects they had seen and the 224 objects they had not seen during the learning phase. Participants were asked to indicate whether or not they had seen the object during the learning phase.

During the learning phase, the factors “choice” and “predictability” were manipulated as follows. For the choice manipulation, participants either chose among two object name cues representing the object they wanted to see (choice condition), or this choice was made for them (no-choice condition). Next, for the predictability manipulation, they either always saw the object corresponding to the selected name (high-predictability condition), or they had a 50% chance to see the object corresponding to the selected name and a 50% chance to see the other object (low-predictability condition). In the low-predictability condition, participants were not explicitly told that the chance of seeing the selected object was 50%. Instead, they were told that “you might have a chance to see the object that you select.” Hence, the whole experiment was a 2 (choice vs. no-choice) × 2 (high predictability vs. low predictability) within-group design.

**Fig. 1:**
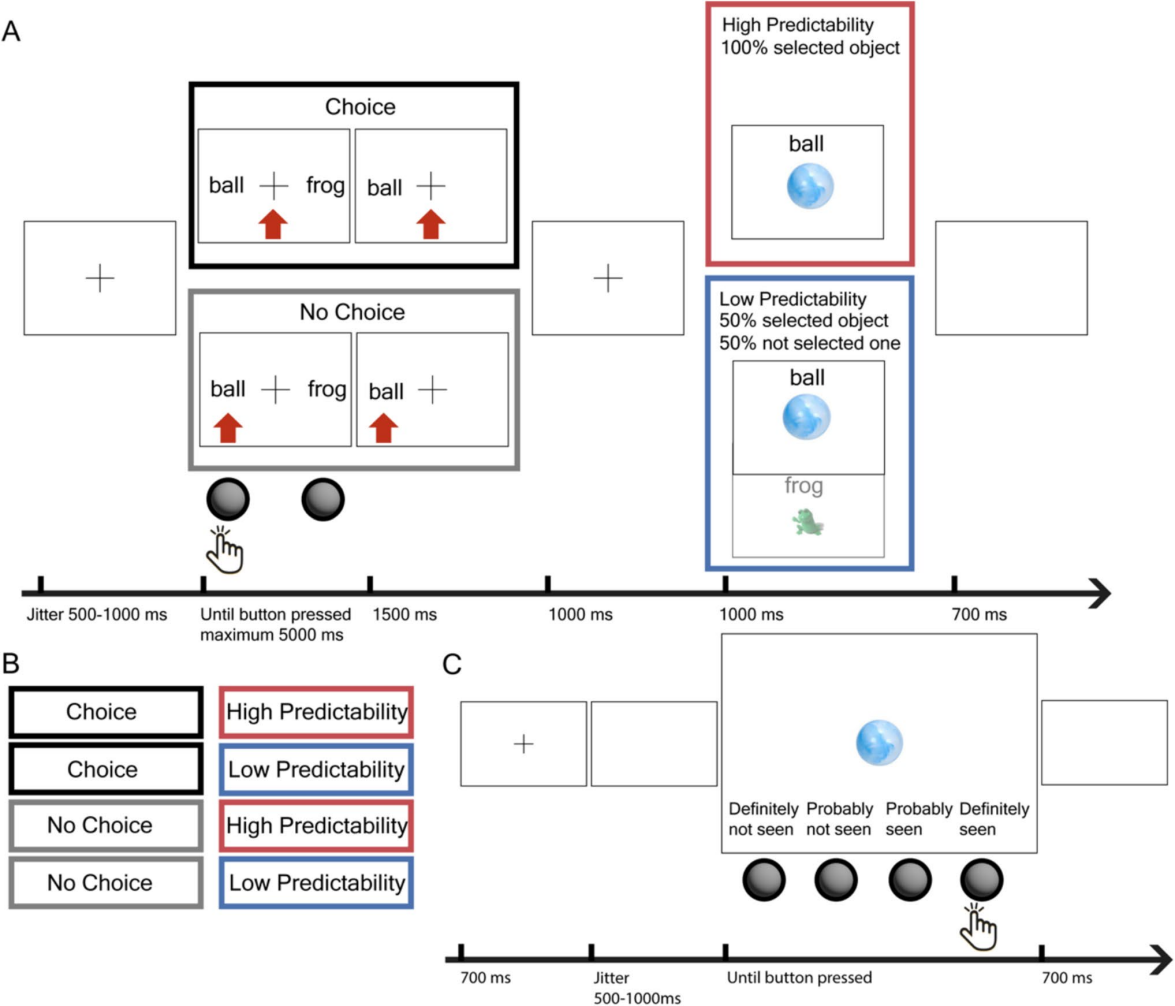
Procedure for Experiment 1. **A.** In the learning phase, each trial started with a fixation cross. Then, in choice conditions (black box), participants would see the red arrow appear in the middle of the screen along with two names of the objects on the left and right side of the screen. They were instructed to choose one of the names by pressing one of the buttons. In no-choice conditions (grey box), participants would see a red arrow on one side of the screen, indicating that they had to press the corresponding button. After this, only the selected name stayed on the screen together with the fixation cross. After the selection stage, in the high-predictability conditions (red box), participants would always see the object that they selected or was selected for them. In the low-predictability conditions (blue box), participants were told that they might have a chance to see the selected object. In practice, this meant that the selected object was presented in 50% of the trials, whereas the not-selected object was presented in the other 50% of the trials. **B.** Overview of the conditions. The choice or no-choice condition was paired with the high-predictability or low-predictability condition. Hence, we had four conditions that would be presented in four separate blocks. The order of the blocks was randomized across participants. **C.** During the memory phase, participants saw a fixation cross followed by an object in each trial. They could press one of the four buttons to indicate whether they have seen the object or not. The possible responses were as follows: “Definitely not seen,” “Probably not seen,” “Probably seen,” and “Definitely seen.” After pressing the button, the object disappears, and they see a blank screen. Then the next trial starts

More specifically, in every trial of the learning phase (Fig. 1A), participants first saw a fixation cross in the center of the screen with presentation time jittered between 500 and 1,500 ms (uniformly distributed). Next, two object names were presented left and right of the fixation cross together with a red up-arrow on the screen for a maximum of 5,000 ms. In the choice condition, participants would see the red up arrow pointing at the fixation cross, indicating that they could select one of the objects that they wanted to see by pressing the button on the left or right. In the no-choice condition, the red up-arrow would already point at one of the object names, and the participants were instructed to press the button on that corresponding side. As soon as the participants pressed the button, only the selected object name would stay on the screen for another 1,500 ms. Next, a white screen was presented (1,000 ms), followed by one of the object pictures with 500 pixels in height presented in the middle of the screen with its name above it (1,000 ms). In the high-predictability condition, participants always saw the object picture that they selected or that was selected for them. In the low-predictability condition, participants had a 50% chance to see the object picture that they selected or that was selected for them, and a 50% chance to see the object picture corresponding to the other object name. In other words: There was low predictability regarding whether they would or would not see the object that was selected before. The trial ended with a white screen (700 ms).

During the learning phase, the four conditions (Fig. 1B) were presented separately in four blocks. The order of the conditions was fully randomized and counterbalanced across participants. Each block contained 56 pairs of objects, which were randomly paired and assigned to one of the conditions beforehand for each participant separately. In line with previous motivational studies, the use of a block design allowed us to keep participants at a stable motivational state across conditions (e.g., Murayama et al., 2010; Voss et al., 2011). Participants were instructed to remember as well as possible the pictures that were made visible in all conditions.

After the four blocks of the learning phase, participants performed a recognition memory test (Fig. 1C). Every trial of the memory test started with a fixation cross presented in the middle of the screen (700 ms), followed by a white screen with a jittered duration between 500 and 1,000 ms (uniformly distributed). Then, an object picture was presented on the screen until a button was pressed. Participants were asked to press a button to indicate whether they had seen the object during the learning phase or not. From left to right, there were four buttons corresponding to “Definitely not seen,” “Probably not seen,” “Probably seen,” and “Definitely seen.” After pressing a button, a white screen would be presented (700 ms), after which the next trial would start. Participants were instructed to perform as accurately as possible.

Before the experiment started, participants underwent a practice session. The practice session consisted of all four learning conditions, which were presented in the same order as the formal learning blocks. There were four trials for each condition and each condition was presented in a different block. Before each block, participants were asked to read the instructions on the screen and summarize them to the experimenter. This was done to ensure that they understood each condition. During the practice session, we showed participants that, if they failed to press a button within 5,000 ms after the object names were presented, a “Too late!” message would be presented on the screen together with one of the object names (1,500 ms). In the choice condition, this object name would be randomly selected for them, while for the no-choice condition, the object name corresponding to the correct selection would stay on the screen. If participants pressed the wrong button in the no-choice condition, they would see the message “Wrong button!” together with the name corresponding to the selection they should have made. In other words, making a wrong response did not affect the selection made for them. There was also a short practice of the memory test, consisting of four trials. In these trials, objects that they saw during the practice learning block were presented. It should be noted that the objects used during the practice session were not included in the actual experiment.

Participants spent between 75 and 90 min in the lab and were paid 15 euros or an equivalent amount of course credits.

#### Data preparation

We prepared data with Python 3.11 (Van Rossum, 2023). Only the objects that were seen by participants were considered for analysis. If a seen object was rated as “probably seen” or “definitely seen” during the memory phase, this memory trial was coded as 1 (accurate). If a seen object was rated as “probably not seen” or “definitely not seen,” this memory trial was coded as 0 (inaccurate). Therefore, for each participant, there were at most 224 memory trials considered for the final analysis. These objects were categorized according to their learning conditions. Objects in the learning trials during which participants did not press a button within the maximum object-name viewing time (5,000 ms) or pressed the wrong button in no-choice learning blocks were excluded (12 out of 12,320 trials in total, 12,308 trials left for analysis).

Furthermore, we also coded how confident participants were that they had seen the object during the learning phase (confident: 1; not confident: 0). Objects that participants indicated to have “definitely seen” or “definitely not seen” in the memory phase were coded as “confident,” and objects indicated as “probably seen” or “probably not seen” were coded as “not confident.” In the end, out of the 12,308 trials included in the analyses, there were 9,676 trials that participants were confident about during the memory phase and 2,632 trials that participants were not confident about. The high portion of confident trials suggested an absence of guessing and a high-quality memory (Meliss et al., 2022)

#### Data analysis

For the primary analyses, we only considered the confident trials (the 9,676 trials participants indicated as “definitely seen” or “definitely not seen” in the memory phase). This was done because when participants responded that the object was “probably seen” or “probably not seen” during the memory test, it might reflect a guess instead of being a signature of actual learning and remembering. This decision was based on a meta-analysis suggesting that intrinsic motivation (e.g., choices, curiosity, or interest) would improve the actual learning performance instead of guesses during the memory test (Cerasoli et al., 2014). In accordance with this argument, it has been suggested that intrinsic motivation could boost learning only for the knowledge that participants were confident about (Galli et al., 2018; Gruber et al., 2014; Meliss et al., 2022; Murphy et al., 2021). Consequently, although we preregistered to include all valid trials for analyses, we decided to perform our analyses on the confident trials alone. The results including only the confident trials are reported in the main text, whereas the results of the preregistered analyses including all trials can be found in Online Supplementary Material 1. It should be noted that both analyses yielded similar results.

The data were modelled with linear mixed-effect modelling (LME) using the *glmer* function of the lme4 package in R (Bates et al., 2015). The main model included accuracy as a binomial dependent variable. The independent variables were choice (yes/no) and predictability (high/low), for both of which we created sum-to-zero contrasts. The main model included the main effects of “choice (yes/no)” and “predictability (high/low),” as well as the interaction effect between “choice (yes/no)” and “predictability (high/low)” as fixed effects. The main model included a full random-effects structure (Barr, 2013; Barr et al., 2013), meaning that a random intercept and random slopes for all effects were included per participant. We fitted the model with 10,000 iterations (5,000 warm-ups) and diagnosed the model with DHARMa (Hartig, 2020).

If the interaction effect between “choice (yes/no)” and “predictability (high/low)” was significant in the main model, we would perform follow-up analyses of simple effects to investigate the choice effect (memory accuracy (choice) – memory accuracy (no-choice)) under different predictabilities. To this end, we modelled the data of the high-predictability and low-predictability conditions separately. We compared the memory test accuracy between choice and no-choice conditions with the *emmeans* function in R (Lenth, 2022). In this way, we were able to detect differences in the choice effect under different predictabilities. On the other hand, with the same procedure as above, we also compared predictability effects (memory accuracy (high predictability) – memory accuracy (low predictability)) under choice or no-choice conditions separately.

Furthermore, to delve deeper into the attenuated choice effect under low predictability, we conducted secondary analyses by separating the trials in the low-predictability conditions into “selected” or “not selected” objects. The “selected” objects were the ones consistent with the name that participants pressed the button on (i.e., selected “ball” and saw the picture “ball”), and the “not selected” objects were the ones under the other name which participants did not press the button on (i.e., selected “ball” but saw the picture “frog”). The details and results of these secondary analyses are described in Online Supplementary Material 2.

## Results

**Fig. 2:**
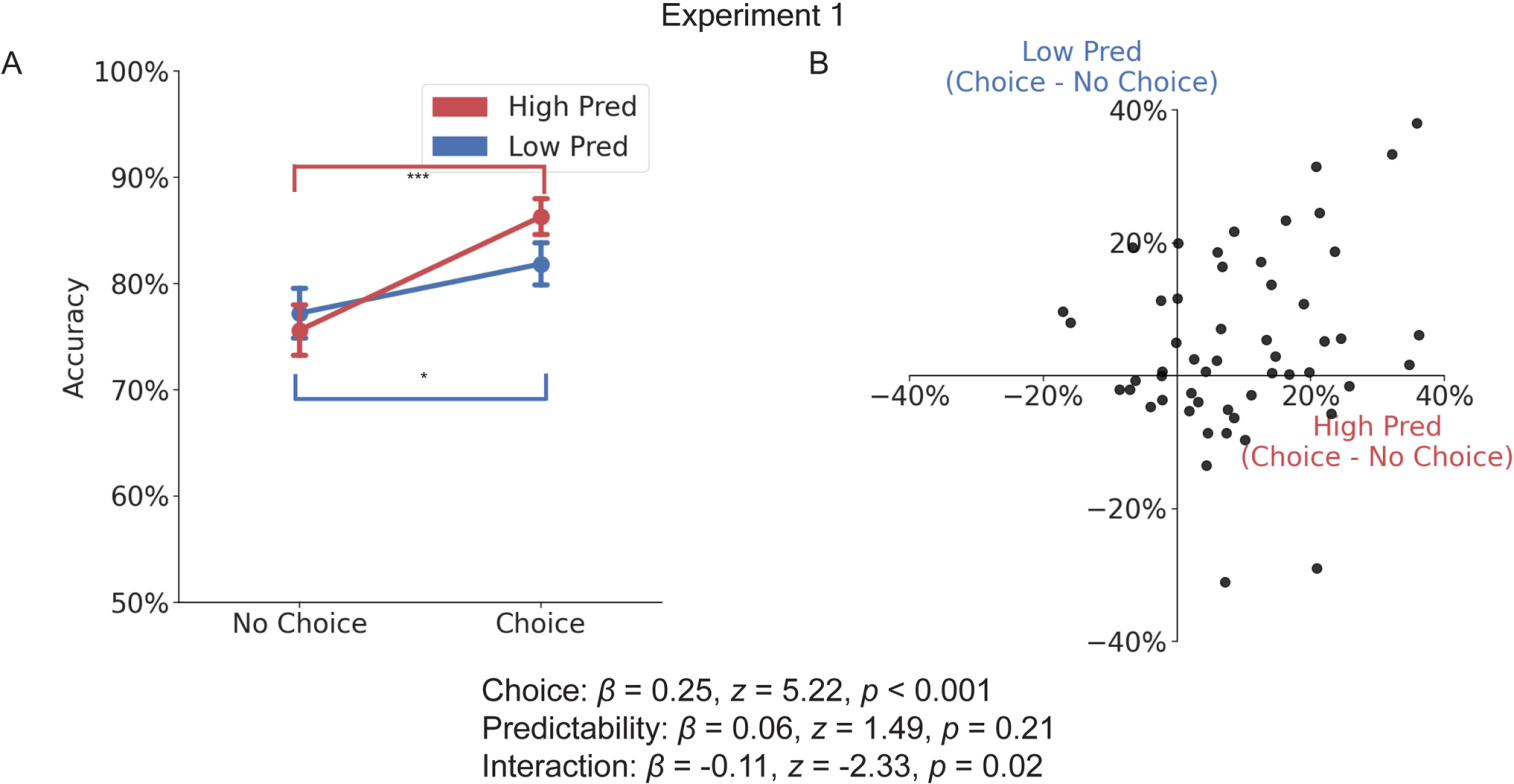
Results for Experiment 1. **A.** For Experiment 1, we found the effect of choice on memory accuracy was significant under different predictabilities, albeit larger for the high-predictability than the low-predictability conditions. Red represents the high-predictability (High Pred) condition and blue represents the low-predictability (Low Pred) condition. The colored lines represent the simple effect comparison (choice – no-choice) under different predictabilities. Asterisks next to the lines indicate the significance of the effects (*** *p* < 0.001; * *p* < 0.05). **B.** Individual variability in the effect of choice for high-predictability conditions (x-axis) compared with low-predictability conditions (y-axis). Each dot represents one participant. For high predictability, most participants showed a positive choice effect (positive x-values). For low predictability, the choice effect appeared to be less strong (indicated by a more even distribution of positive and negative y-values). This reflects the reported interaction between choice and predictability on memory accuracy

For the primary analysis (Fig. 2A and B), we found a main effect of choice (*β* = 0.25, *z* = 5.22, *p* < 0.001) on memory accuracy. This indicated that when people could choose, their memory accuracy was higher (*M* = 84.3%, *SD* = 12.6%) than in no-choice conditions (*M* = 76.6%, *SD* = 16.2%). In contrast, there was no significant main effect of predictability (*β* = 0.06, *z* = 1.49, *p* = 0.21), indicating that there was no difference in memory accuracy under high predictability (*M* = 81.4%, *SD* = 13.3%) or low predictability (*M* = 79.7%, *SD* = 14.6%).

However, we also found a significant interaction effect of choice and predictability on memory accuracy (*β* = ˗0.11, *z* = ˗2.33, *p* = 0.02). This means that, under the high-predictability conditions, memory accuracy was significantly higher in the choice condition (*M* = 86.3%, *SD* = 12.5%) than in the no-choice condition (*M* = 75.6%, *SD* = 17.4%; *β* = 0.71, *z* = 5.28, *p* < 0.001). The same was true for the low-predictability conditions. The memory accuracy was higher in the choice condition (*M* = 81.9%, *SD* = 14.8%) than in the no-choice condition (*M* = 77.21%, *SD* = 17.19%; *β* = 0.29, *z* = 2.28, *p* = 0.02). However, the difference in memory accuracy between choice and no-choice conditions under high predictability was larger than this difference under low predictability (Fig. 2A). Additionally, there was a difference in memory accuracy between high- and low-predictability conditions when people could choose (*β* = 0.34, *z* = 2.58, *p* = 0.01), but not when people could not choose (*β* = −0.08, *z* = −0.71, *p* = 0.48).

To summarize, we found an effect of choice on memory accuracy for both the high- and the low-predictability condition. However, this effect appeared to be stronger for the high-predictability condition compared with the low-predictability condition (Fig. 2A).

## Experiment 1: Discussion

In the first experiment, we found a significant main effect of choice on memory accuracy. Participants showed better memory for objects when they had the opportunity to choose than when they could not choose. More interestingly, we found a noteworthy significant interaction effect on memory accuracy between the manipulated factors of choice and predictability. The improvement in memory accuracy attributed to the choice effect was somewhat diminished, but still statistically significant under low-predictability conditions.

These findings provide evidence that supports both interpretations of the choice effect. On the one hand, the existence of the choice effect under both high and low predictability suggested that choices were by themselves regardless of the consequences (Murty et al., 2015). On the other hand, the interaction effect between choice and predictability, indicating that the choice effect diminished under low-predictability conditions, suggested that choices aided in learning because they enabled individuals to predict the outcomes associated with their choices (Katzman & Hartley, 2020).

However, these findings left open questions behind. In the current setup, within the low-predictability conditions, half of the objects that participants saw were not the ones they selected, thus creating an inconsistency between the outcome and predictions associated with their choices. This inconsistency might cause a worse memory of the objects (Frankenstein et al., 2020). Secondary analyses were carried out to ascertain whether the presence of these not-selected objects was the sole cause for the dampening of the choice effect (see Online Supplementary Material 2). We separated the objects under low predictability into “selected” and “not selected.” It was revealed that the choice effect on memory accuracy for both selected and non-selected objects in the low-predictability condition was significant and comparable, albeit noticeably attenuated compared to the high-predictability condition. In other words, this reduction in the choice effect was not limited to not-selected objects, it also applied to selected objects.

In summary, based on the findings of Experiment 1, we can conclude that the choice effect on memory was attenuated when participants could not predict the outcome of choices, regardless of whether the outcome was consistent with their choices or not. Thus, the choice effect seems to be related to both the satisfaction of having a choice and the (sensorimotor) consequences that the choice was associated with.

## Experiment 2

A conspicuous distinction nevertheless existed between the choice and no-choice conditions. Making a choice is time-consuming (Online Supplementary Material 3). As a consequence, participants viewed the object names (object-name viewing time) longer in the choice than in the no-choice condition, and this effect of choice on object-name viewing time was larger under high than low predictability (Online Supplementary Material 3, Fig. S3). Experiment 2 is designed to investigate this fact in more detail since these differences in viewing times might explain the reported effects of choice and predictability on memory accuracy.

To rule out this possibility, we conducted Experiment 2. To this end, we adjusted the paradigm such that (1) the amount of time that the object names were shown to the participants was controlled and kept constant between conditions, and (2) ensured that participants always read both object names before seeing the objects themselves. The latter was done by separately presenting each object name for a fixed amount of time before participants were asked to make their choice.

If the reported effects of choice and predictability on memory accuracy are annihilated when controlling for object-name viewing time, it might be the case that these object names function similarly to cues that facilitate memory (e.g., Thomson & Tulving, 1970). Consequently, longer exposure to these cues might result in better memory of the affiliated pictures. However, if we find similar effects on memory accuracy to those reported for Experiment 1, the main effect of choice on memory accuracy and the interaction between choice and predictability on memory accuracy is likely not driven by object-name viewing time.

### Methods

#### Participants

For Experiment 2, we recruited 56 participants (average age = 22.27 ± 2.42 years; 42 females, 14 males) with the same criteria as Experiment 1.

#### Procedure

In Experiment 2, a similar paradigm was used as in Experiment 1. We made a few adaptations to control for differences in object-name viewing time between conditions.

**Fig. 3:**
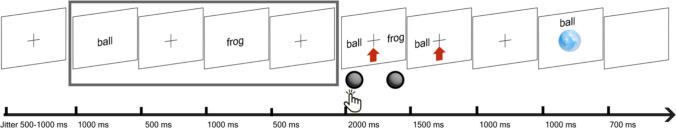
Procedure for Experiment 2 (choice/high-predictability trial as an example). The paradigm from Experiment 1 is adapted to control the amount of time that participants viewed the names corresponding to the objects. After the jittered fixation, participants would view the names of the objects one at a time for 1,000 ms, followed by 500 ms of fixation. Since both object names were shown in the middle of the screen one by one, we ensured that participants would perceive and process both object names (see gray frame). Afterwards, participants were presented with both object names again and were asked to respond by pressing one of the buttons (2,000 ms). Crucially, both object names were on the screen for the full 2,000 ms. The rest of the experiment remained the same as Experiment 1

Instead of the choice screen shown right after the jittered fixation cross, participants would first see one object name at a time. For example, the object names “ball” and “frog” were each presented for 1,000 ms (one after the other), separated by a 500-ms fixation cross (Fig. 3). Following the second object name, another 500-ms fixation cross was presented. Next, the choice screen with the object names and the red up-arrow is presented for a fixed duration of 2,000 ms, during which participants had to press a button. In this way, we ensured that both object names were visible for a fixed amount of time (instead of disappearing after the button press, as was the case in Experiment 1). The rest of the trial remained the same as for Experiment 1.

The memory phase was the same as in Experiment 1. For Experiment 2, participants spent between 100 and 110 minutes in the lab and were paid 20 euros for participation or an equivalent amount of course credits.

#### Data analysis

The data were preprocessed in the same way as for Experiment 1. Of data from all the participants, we eliminated 136 (out of 12,544) trials because of incorrect or too-late responses. Out of the total of 12,408 valid trials, there were 10,142 trials for which participants were confident (responded with “definitely seen” or “definitely not seen”), reflecting high-quality memory in the current experiment (Meliss et al., 2022). As for Experiment 1, we omitted the unconfident trials (responded with “probably seen” or “probably not seen”) and conducted a main 2 (choice or no-choice) × 2 (high predictability or low predictability) LME analysis on memory accuracy. As for Experiment 1, the results including all trials are reported in Online Supplementary Material 1.

The same secondary analyses as mentioned for Experiment 1 were conducted on the data of Experiment 2. These results can be found in Online Supplementary Material 2.

## Results

**Fig. 4:**
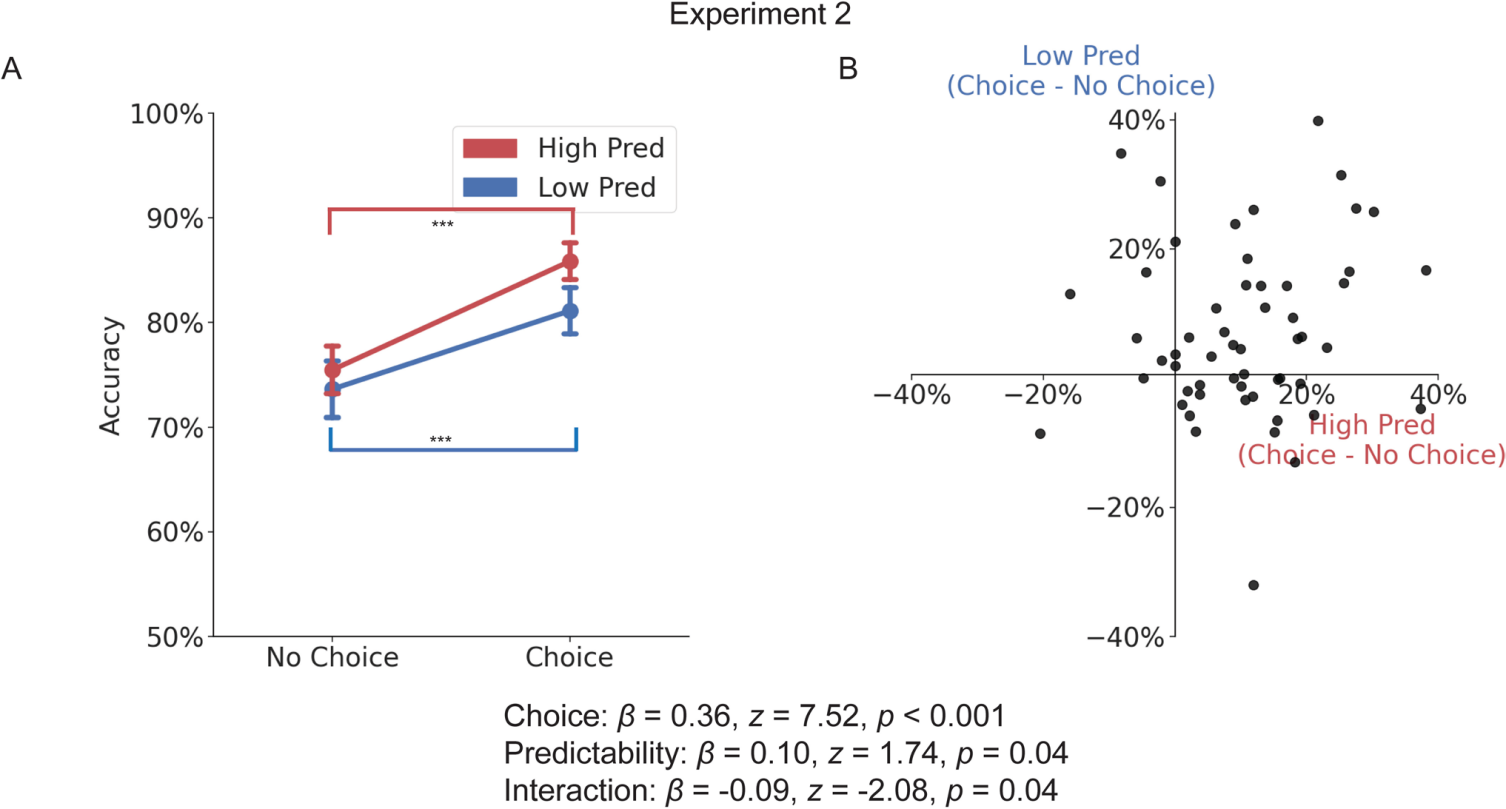
Results for Experiment 2. **A.** For Experiment 2, we found similar results to those of Experiment 1. The effect of choice on memory accuracy was significant under different predictabilities, albeit larger for the high-predictability than the low-predictability conditions. Red represents the high-predictability (High Pred) condition and blue represents the low-predictability (Low Pred) condition. The colored lines represent the simple-effect comparison (choice – no-choice) under different predictabilities. Asterisks next to the lines indicate the significance of the effects (*** *p* < 0.001; * *p* < 0.05). **B.** Individual variability in the effect of choice for high-predictability conditions (x-axis) compared with low-predictability conditions (y-axis). Each dot represents one participant. For high predictability, most participants showed a positive choice effect (positive x-values). For low predictability, the choice effect appeared to be less strong (indicated by a more even distribution of positive and negative y-values). This reflects the reported interaction between choice and predictability on memory accuracy

In Experiment 2, we replicated the main findings of Experiment 1 (Fig. 4A and B). First, we found a main effect of choice (*β* = 0.36, *z* = 7.52, *p* < 0.001) on memory accuracy. This indicated that when people could choose, their memory accuracy was higher (*M* = 83.7%, *SD* = 13.9%) than in no-choice conditions (*M* = 74.8%, *SD* = 16.3%). Second, there was also a significant main effect of predictability (*β* = 0.10, *z* = 1.74, *p* = 0.04). This indicated that people remembered objects better under high predictability (*M* = 80.8%, *SD* = 14.0%) than under low predictability (*M* = 77.7%, *SD* = 16.9%). Last but not least, as in Experiment 1, there was also a significant interaction effect of choice and predictability on memory accuracy (*β* = −0.09, *z* = −2.08, *p* = 0.04).

Furthermore, follow-up analyses with *emmeans* yielded similar results to those found in Experiment 1. When people could predict the outcome of their choices well (high predictability), we found that the accuracy in the choice condition (*M* = 85.9%, *SD* = 13.3%) was significantly higher than in the no-choice condition (*M* = 75.5%, *SD* = 16.9%; *β* = 0.89, *z* = 6.99, *p* < 0.001). When people could not predict the outcome of their choices well (low predictability), we found that the accuracy under the choice condition (*M* = 81.1%, *SD* = 16.6%) was also significantly higher than the no-choice condition (*M* = 73.6%, *SD* = 20.0%; *β* = 0.53, *z* = 4.17, *p* < 0.001). Under the choice condition, people remembered objects better when there was high predictability compared with low predictability (*β* = 0.37, *z* = 2.82, *p* = 0.005), but this difference was not present in the no-choice condition (*β* = 0.02, *z* = 0.12, *p* = 0.91).

To conclude, in Experiment 2, we found an effect of choice in both the high and the low-predictability conditions. As in Experiment 1, this effect appeared to be stronger for the high-predictability condition compared with the low-predictability condition (Fig. 4A).

## Experiment 2: Discussion

In Experiment 2, we employed a similar paradigm to that in Experiment 1. However, in each trial, the object names were presented one at a time before the choice screen. Also, the object-name viewing time remained the same for 2,000 ms across all trials (Fig. 3). We replicated the main results on memory accuracy from Experiment 1. That is, the beneficial effect of choice on memory accuracy always stayed statistically significant under both high and low predictabilities. At the same time, the choice effect on memory was notably diminished under low predictability.

In conclusion, in Experiment 2, the main results were replicated, suggesting that choice would always help with memory, even when participants could not predict. At the same time, this choice effect on memory was attenuated when the choice outcome predictability was low. Interestingly, in Experiment 2, the choice effect on memory accuracy got smaller for non-selected objects, but not for selected objects under low predictability. This was different from Experiment 1 (see Online Supplement Material 2).

## General discussion

In a set of two experiments, we aimed to gain a better understanding of the beneficial effect of choice on memory. For this purpose, we designed a well-controlled experimental paradigm to test the choice effect on memory accuracy under different predictabilities. On the one hand, if the choice effect on accuracy was the same for high and low predictabilities, we would conclude that choices are motivating by themselves regardless of the outcome (Ding et al., 2021; DuBrow et al., 2019; Murty et al., 2015; Rotem-Turchinski et al., 2019). On the other hand, if the choice effect is solely present under high predictability, this would suggest that choices are beneficial for learning because they facilitate predictive processing (Cockburn et al., 2014; Desantis et al., 2011; Gureckis & Markant, 2012; Katzman & Hartley, 2020; Luo et al., 2022; Markant et al., 2014; Meng & Ma, 2015; Moore & Haggard, 2008; Schneider et al., 2018; Sharot & Sunstein, 2020; Voss et al., 2011). Based on our results, we found evidence for both explanations. We found a facilitatory effect of choice on memory accuracy for both high- and low-predictability conditions. Yet, the choice effect on memory accuracy was markedly smaller under low predictability than under high predictability. These results together support both hypotheses we raised. In essence, choices are internally motivating by themselves, but they also enhance memory by fostering predictive processing.

## Choices facilitate learning because they afford intrinsic motivation

In both experiments we found that participants remembered objects better when they could choose than when they could not choose. This main effect of choice on memory accuracy maintained statistical significance throughout both high and low predictabilities, even for the objects that were not selected in low-predictability conditions (Fig. S2, Online Supplemental Material 2). This finding substantiates the hypothesis that choices (partially) facilitate learning due to the intrinsic motivation associated with the opportunity to choose.

According to the self-determination theory (SDT), choices enhance the feeling of autonomy, one of the three fundamental needs in intrinsic motivation for learning (Ryan & Deci, 2020). Autonomy is defined as the feeling of ownership and freedom of one’s actions. One of the core arguments in SDT for education is that students would feel more engaged and self-related in learning when their need for autonomy is satisfied. Students would even show higher emotional arousal during learning when their need for autonomy is supported (Streb et al., 2015). Even when the subsequent outcome did not align with the choices that participants made, having the opportunity to choose by itself already contributed to the facilitation of learning. This is in line with previous studies indicating that even inconsequential choices boost memory formation (Ding et al., 2021; Murty et al., 2015; Rotem-Turchinski et al., 2019). Furthermore, having control over one’s own action is also referred to as the sense of agency, which is closely linked to dopaminergic circuitry associated with reward and motivational processing. This sense of agency has been found to increase motivation intrinsically (Ashby et al., 2024). Notably, Bobadilla-Suarez et al. (2017) found that people would even give up rewards to retain agency when making choices. They would pay in order to maintain control of their own payoff, supporting the intrinsic psychological values of choices. In conclusion, the inherently satisfying feelings that choices bring for people can in themselves enhance learning processes and performances.

## Choices enhance learning by facilitating prediction of outcomes

In both experiments, we found that the choice effect on memory accuracy was attenuated under low predictability compared with high predictability. This implies that part of the advantage that choices bring for learning is due to the more active prediction that choices elicit.

To start with, the act of choosing transforms the information process from passive perceiving to active predicting. Our brains are not old-fashioned computers that can only take passive inputs and produce responses. On the contrary, our brains are active inference agents that constantly predict upcoming events in the surrounding environments (Friston, 2010; Friston et al., 2013, 2016). Choices could enhance active inference, leading to a facilitation of reducing prediction error between predicted states and perceived information (Friston et al., 2013). This would explain that when participants could not accurately predict the outcome of their choices, their prediction was violated. Under this circumstance, the sense of agency (i.e., autonomy, the feeling of control when having the chance to choose) would also be attenuated (Friston et al., 2013). The loss of sense of agency also results in a feeling of losing control over the situation. Such uncontrollability would cause frustration and learned helplessness, leading to impaired motivation and learning performance (Mineka & Hendersen, 1985; Seligman, 1972). In such a situation, students could sense that making choices is not “useful,” leading to a loss of controllability and diminishing of the beneficial effect of choice on learning (Katz & Assor, 2007). In addition, some studies posit that choices would lead to a distortion of the information value after it was perceived. When people are choosing, they might feel that information is more valuable than when they could not choose (DuBrow et al., 2019; Izuma et al., 2013; Meng & Ma, 2015; Sharot & Sunstein, 2020). For example, it was found that people would have a higher expectation of success in a cognitive task that they chose, shown by both behavioral and electrophysiological evidence (Meng & Ma, 2015). As a result, the choice effect on memory accuracy became smaller under low predictability.

Since people would more actively predict upcoming information when they could choose, they could also coordinate their attention beforehand. This was supported by previous findings showing that even if people could only control *when* to adjust their attention to the next object, memory would already be boosted (Markant et al., 2014). This attentional tuning supports memory encoding and retention before information even appears (Gureckis & Markant, 2012). In a study conducted by Luo et al. (2022), it was suggested that attention preparation would only occur when the outcome of the choice was predictable. In their experimental setting, participants were more concentrated and exhibited faster reaction times in the subsequent attentional task when they had chosen and could predict the background picture for each trial. However, when participants were unable to predict the consequences of their choices for the background pictures in the same paradigm, the reaction time of the subsequent task was not accelerated by making active choices.

Moreover, previous research has shown that choice behavior facilitates the minimization of surprise and the maximization of precision in processing reward-related information (Schwartenbeck et al., 2015a, 2015b). Specifically, Schwartenbeck et al. (2015a) indicated that the dopaminergic midbrain, a key component of the reward system involved in motivational states, plays a central role in encoding expected certainty or precision about desired outcomes, supporting the minimization of surprise. In addition, prefrontal regions, commonly associated with attentional control, have also been implicated in precision weighting and predictive processing when making choices. These findings provide a theoretical bridge to our current results, suggesting that active choices enhance memory encoding not only by being intrinsically motivating, but also by facilitating attentional and informational preparation for upcoming visual input. These findings can be translated to the experiments described here. If our participants chose “ball” by themselves, they might have already tuned their attention to the state of seeing a ball picture later. However, if the “ball” was chosen for them, this prior prediction and attention tuning might be less active (Fig. 1).

In summary, in the current experiment, we found that the choice facilitatory effect on memory diminished under low predictability. This finding supports the hypothesis that choice improves learning by enhancing prediction over the consequences. Two potential explanations for this attenuation of the choice effect emerge. On the one hand, choices brought a more active prediction of future information so that people would coordinate their attention in advance. On the other hand, choices evoked a sense of lower prediction error between the choice and the perceived information.

## Combining self-determination theory and active inference to explain the beneficial effect of choices in memory

Our results support both hypotheses raised in the *Introduction*. First, choices enhanced learning through their inherent value, as evidenced by robust effects of choice on memory accuracy across all conditions. Second, choices improved learning by facilitating the prediction of subsequent outcomes, consistent with the attenuation of the choice effect when predictability was low.

Our main effects and interactions speak to the validity of both self-determination theory and active inference formulations of choice behavior and learning. Perhaps this is not surprising, given their shared emphasis on intrinsic motivation. In SDT, intrinsic motivation to learn is considered a fundamental psychological need (Ryan & Deci, 2020). In active inference, choices are based upon the expected information gain and expected utility of outcomes consequent on a choice or policy, often framed as intrinsic and extrinsic value, respectively (Schwartenbeck et al., 2019; van Lieshout et al., 2025). The intrinsic motivation (expected information gain) underscores the epistemic value and the drive of exploratory, curiosity-driven or information-seeking behavior in developmental neurorobotics and cognitive neuroscience (Barto et al., 2013; Oudeyer & Kaplan, 2007; Parr & Friston, 2019; Schmidhuber, 2010; Schwartenbeck et al., 2019). Importantly, this formulation resolves the exploration-exploitation dilemma: intrinsic motivation is precisely the drive to reduce uncertainty, thereby enabling more efficient learning. From this perspective, it is not surprising that allowing participants to make intrinsically motivated choices promotes memory, since such choices are designed to maximize information gain (Schwartenbeck et al., 2019).

A more subtle implication is that the opportunity to choose may itself promote learning. Anticipating the chance to learn can lead participants to update their (Bayesian) beliefs and increase the precision of their predictions towards information, instantiating a particular attentional adjustment (Feldman & Friston, 2010; Hohwy, 2012; Limanowski, 2022). This attentional adjustment predisposes them to learning, effectively facilitating memory encoding and later recall. In SDT terms, this mechanism provides a computational account of the “feeling of autonomy”: having the ability to choose not only fulfils a psychological need but also optimally prepares the learner to update (Bayesian) beliefs and acquire new knowledge.

## Consistency between choice and outcome can partially explain the choice effect on learning

When we dissected the condition of low predictability into selected and not-selected objects, we found different patterns of results in the two experiments (Online Supplementary Material 2, Figs. S2C and D). To be more specific, the choice effect on memory accuracy diminished under low predictability for both selected and not-selected objects in Experiment 1. In contrast, in Experiment 2, the choice effect on memory accuracy did not diminish for selected objects under low predictability.

This discrepancy may be attributed to the fact that in Experiment 2, participants were guaranteed to view both object names for a fixed amount of time. This might elicit predictions regarding both objects in the low-predictability conditions, causing confusion and perhaps even false memory. This is consistent with the mechanism of proactive interference and divided attention since more encoded cues might cause a higher cognitive load (Jacoby et al., 2010; Kane & Engle, 2000). Hence, memory accuracy for selected objects in the low-predictability condition might be reduced when participants had no choice. In contrast, when participants were choosing objects by themselves, even under low predictability, they might have constructed a stronger anticipation of the selected object (Meng & Ma, 2015). As a consequence, memory under the choice/low-predictability condition might not be confused by these multiple predictions. Therefore, the choice effect on memory accuracy for selected objects under low predictability was larger in Experiment 2 compared with Experiment 1. Based on this result, we could demonstrate that when the upcoming information fits the prediction, the choice effect would not be attenuated. These findings provided more evidence for the predictive processing perspective of the choice facilitatory effect on learning and memory.

Meanwhile, we found that memory performance was worse for the low-predictability condition compared with the high-predictability condition only when participants were making choices freely. Importantly, this is consistent with previous findings indicating that a mismatch between prediction and perceived information diminishes information encoding (Csink et al., 2021; Frank et al., 2022) but contrasts with other work reporting enhanced visual representation or memory under the prediction-violation condition compared to the prediction-consistent condition (Filimon et al., 2020; Richter et al., 2018). There are two explanations for this discrepancy in the direction of the predictability effect on memory or visual representation. First, in our design, participants were told that they might or might not see the object that they chose or was chosen for them. In our design, participants were aware that outcomes could deviate from their choices (Piray & Daw, 2024), in other words, expected violation. In such contexts, participants could down-weight the perceived information after making the choice, which can impair encoding due to increased cognitive conflict or reduced attentional alignment (Luo et al., 2022; Markant et al., 2014a). Second, the diminishing effect of low predictability on memory encoding under the choice condition may also be attributed to the differential influence of information mismatch on memory processes depending on task demands (Frank & Kafkas, 2021; Kafkas & Montaldi, 2018). It was found that expected information tends to enhance familiarity, whereas unexpected information is more likely to support recollection (Kafkas & Montaldi, 2018). Given that the current task primarily tapped into familiarity-based recognition, the observed memory-encoding benefit for expected information is consistent with this notion. In summary, in the current setting, lower predictability diminished memory encoding but only when people were making active choices.

## Preference was not the only reason for the choice effect on learning

Additionally, there might be a possibility that the choice effect on memory accuracy is driven by participants’ preferences. When participants had the opportunity to choose, they would most likely choose the objects that they preferred (Verdugo et al., 2023). This was not the case in the no-choice conditions: participants would be allocated to one of the objects randomly and there was no opportunity to follow their preferences. Hence, the beneficial effect of choice on memory might also be caused by participants’ higher preference for chosen compared with not chosen objects. However, our results mostly rule out this possibility, since we found that choices also improved learning when we focused solely on objects that were not the ones that they selected and thus preferred (Online Supplementary Material, Fig. S2D). If preference was the driving factor behind the observed effect of choice on memory accuracy, we would expect that the choice effect on memory accuracy would disappear for the not-selected objects under low predictability. However, we found that choices enhanced memory accuracy for all conditions.

## Future directions

Although the current study showed that the beneficial effect of choice on memory is modulated by the predictability of choice outcomes, there is still more to be investigated with this topic. For example, Luo et al. (2022) found that the facilitatory effect of choices on attention allocation (measured by reaction time in an attentional task) completely disappeared when the participants had inaccurate predictions over the outcome of the choices. On the contrary, in the current study, the choice effect on memory remained significant but was smaller under low predictability. However, it is hard to get a better understanding of these partially conflicting findings with only behavioral measures. Therefore, neuroimaging studies are required to delve deeper into the mechanisms of how choices facilitate learning by modulating predictions. It would be intriguing to implement functional magnetic resonance imaging (fMRI) scanning along with the same paradigm in the future. For instance, based on our findings, it might be the case that having a choice is both motivating in itself and helps us to better predict future outcomes (i.e., by tuning our attention to upcoming information). Considering both perspectives, we hypothesize that the connectivity between the prefrontal cortex and striatum would be stronger in the choice than no-choice condition (Leotti et al., 2010; Murty et al., 2015), and this choice effect on brain connectivity would be attenuated by low predictability.

## Conclusion

In conclusion, the current study showed that choices can help people to learn better under both high and low predictabilities of the choice outcomes. However, the choice facilitatory effect on memory was diminished when the outcomes of the choices could not be accurately predicted. Our findings demonstrate that an opportunity to choose will help with learning since choices have satisfactory values by themselves. At the same time, the predictability of upcoming information also modulates the facilitatory effect of choice on learning. This indicates that the choice effect on learning is partially dependent on prediction processes. These results can easily be adapted to educational situations. As an illustration, in the context of learning to play the piano, instructors may inquire about students’ musical preferences and tailor educational plans accordingly. This personalized approach, rooted in individual choices, holds the potential to enhance the likelihood of success in learning and education.

## Supplementary Information

Below is the link to the electronic supplementary material.

Supplementary file1 (DOCX 3039 KB)

Supplementary file2 (DOCX 6101 KB)

Supplementary file3 (DOCX 1766 KB)

## Data Availability

All data and code used for stimulus presentation and analyses of both experiments are freely available on the Donders Repository (10.34973/rn1x-d471).
