# Supplementary material for: Autonomy in learning: Predictability modulates the beneficial effect of choice on memory
Source: Mem Cognit. 2026 Feb 25;54(6):2197–211. doi: 10.3758/s13421-026-01863-9 (PMC13461742; doi:10.3758/s13421-026-01863-9)
Supplement: Supplementary file 1 [file 13421_2026_1863_MOESM1_ESM.docx]

**Supplement 1: Primary analysis with all trials**

Following the preregistration, we conducted the same primary analysis as reported in the main text on all trials instead of only the confident trials. We did so for both Experiment 1 and Experiment 2.

**Results**

As reported in the main text, we found an interaction between choice and predictability on memory accuracy in Experiment 1 and 2 when only confident trials were included. We also found this interaction when including all trials in Experiment 1, but the interaction effect did not reach significance in Experiment 2 (see Table S1, Figure S1).

A possible explanation for not finding this effect in Experiment 2 is that the effect of choice was attenuated when also considering trials that participants were not confident about. This is in line with previous work suggesting that intrinsic motivators (e.g., choice or curiosity) boost our learning, but only for information that participants were confident about (Galli et al., 2018; Gruber et al., 2014; Meliss et al., 2022; Murphy et al., 2021).

Table S1 Linear Mixed Effect Model from Primary Analyses

|  | **Experiment1** | **Experiment2** |
| --- | --- | --- |
| **Memory accuracy: all trials** | | |
| Choice | *β* = 0.23, *z* = 6.10, *p* < 0.001 | *β* = 0.29, *z* = 7.38, *p* < 0.001 |
| Predictability | *β* = 0.06, *z* = 1.84, *p* = 0.16 | *β* = 0.06, *z* = 1.79, *p* = 0.06 |
| Choice * Predictability | *β* = -0.09, *z* = -2.47, *p* = 0.01 | *β* = -0.06, *z* = -1.57, *p* = 0.12 |
| *Follow-up t-tests:* |  |  |
| *Choice – No choice (High Pred)* | *β* = 0.64, *z* = 6.04, *p* < 0.001 | *β* = 0.69, *z* = 7.04, *p* < 0.001 |
| *Choice – No choice (Low Pred)* | *β* = 0.29, *z* = 2.81, *p* = 0.005 | *β* = 0.46, *z* = 4.00, *p* = 0.001 |
| *High Pred - Low Pred (Choice)* | *β* = 0.31, *z* = 2.81, *p* = 0.005 | *β* = 0.27, *z* = 2.47, *p* = 0.01 |
| *High Pred - Low Pred (No Choice)* | *β* = -0.05, *z* = -0.52, *p* = 0.606 | *β* = 0.04, *z* = 0.37, *p* = 0.71 |
| **Memory accuracy: confident trials (as also reported in the main text)** | | |
| Choice | *β* = 0.25, *z* = 5.22*, p < 0.001* | *β* = 0.36, *z* = 7.52, *p* < 0.001 |
| Predictability | *β* = 0.06, *z* = 1.49, *p* = 0.21 | *β* = 0.10, *z* = 1.74, *p* = 0.04 |
| Choice * Predictability | *β* = -0.11, *z* = -2.33, *p* = 0.02 | *β* = -0.09, *z* = -2.08, *p* = 0.04 |
| *Follow-up t-tests:* |  |  |
| *Choice – No choice (High Pred)* | *β* = 0.71, *z* = 5.28, *p* < 0.001 | *β* = 0.89, *z* = 6.99, *p* < 0.001 |
| *Choice – No choice (Low Pred)* | *β* = 0.29, *z* = 2.28, *p* = 0.02 | *β* = 0.53, *z* = 4.17, *p* < 0.001 |
| *High Pred - Low Pred (Choice)* | *β* = 0.34, *z* = 2.58, *p* = 0.01 | *β* = 0.37, *z* = 2.82, *p* = 0.005 |
| *High Pred - Low Pred (No Choice)* | *β* = -0.08, *z* = -0.71, *p* = 0.48 | *β* = 0.02, *z* = 0.12, *p* = 0.91 |

Note: Memory accuracy ~ choice * predictability + (1 + choice * predictability|subject)

Table S2 Descriptive statistics for memory accuracy based on primary analysis

|  |  | **Experiment 1 M ± SD** | **Experiment 2 M ± SD** |
| --- | --- | --- | --- |
| **Accuracy: all trials** | | | |
| Choice main effect | Choice | 79.3 % ± 11.9% | 79.9% ± 13.6% |
|  | No choice | 71.4% ± 14.3% | 70.9% ± 15.4% |
| Predictability main effect | High pred | 76.4% ± 11.8% | 77.0% ± 13.5% |
|  | Low pred | 74.7% ± 13.8% | 73.8% ± 15.9% |
| Follow-up comparison | *Choice (High pred)* | *81.9% ± 11.6%* | *82.1% ± 13.1%* |
|  | *Choice (Low pred)* | *76.7% ± 14.8%* | *77.5% ± 16.5%* |
|  | *No choice (High pred)* | *70.9% ±15.2%* | *71.9% ±16.0%* |
|  | *No choice (Low pred)* | *71.8% ± 15.6%* | *69.9% ± 18.4%* |
| **Accuracy: confident trials (main text)** | | | |
| Choice main effect | Choice | 84.3% ± 12.6% | 83.7% ± 13.9% |
|  | No choice | 76.6% ± 16.2% | 74.8% ± 16.3% |
| Predictability main effect | High pred | 81.4% ± 13.3% | 80.8% ± 14.0% |
|  | Low pred | 79.7% ± 14.6% | 77.7% ± 16.9% |
| Follow-up comparison | *Choice (High pred)* | *86.3% ± 12.5%* | *85.9% ± 13.3%* |
|  | *Choice (Low pred)* | *81.9% ± 14.8%* | *81.1% ± 16.6%* |
|  | *No choice (High pred)* | *75.6% ± 17.4%* | *75.5% ± 16.9%* |
|  | *No choice (Low pred)* | *77.21% ± 17.19%* | *73.6% ± 20.0%* |

***Figure S1. Main results including all trials.***

***A.*** *Experiment 1. Even including all trials in Experiment 1, we still found the significant main effect of choice and interaction between choice and predictability on memory accuracy. Participants remember objects better when they could choose than when they could not choose. Meanwhile, the choice effect was stronger under high than low predictability. These results are the same as the results including only the confident trials, as reported in the main text.*

***B.*** *Experiment 2. When we controlled for object viewing time in Experiment 2, the interaction between choice and predictability was not found in memory accuracy including all trials. However, there was still a main effect of choice on memory accuracy. This is perhaps unsurprising, given that non-confident guesses likely reflect random guesses instead of actual learning, hereby indicating a low quality of memory. As suggested by previous studies (Cerasoli et al., 2014; Meliss et al., 2022), low quality memory might be less boosted by intrinsic motivators (i.e. choices).*

**References**

Cerasoli, C. P., Nicklin, J. M., & Ford, M. T. (2014). Intrinsic motivation and extrinsic incentives jointly predict performance: a 40-year meta-analysis. *Psychological Bulletin*, *140*(4), 980-1008. https://doi.org/10.1037/a0035661

Galli, G., Sirota, M., Gruber, M. J., Ivanof, B. E., Ganesh, J., Materassi, M., Thorpe, A., Loaiza, V., Cappelletti, M., & Craik, F. I. M. (2018). Learning facts during aging: the benefits of curiosity. *Experimental Aging Research*, *44*(4), 311-328. https://doi.org/10.1080/0361073X.2018.1477355

Gruber, M. J., Gelman, B. D., & Ranganath, C. (2014). States of curiosity modulate hippocampus-dependent learning via the dopaminergic circuit. *Neuron*, *84*(2), 486-496. https://doi.org/10.1016/j.neuron.2014.08.060

Meliss, S., van Reekum, C., & Murayama, K. (2022). Broad Brain Networks Support Curiosity-Motivated Incidental Learning Of Naturalistic Dynamic Stimuli With And Without Monetary Incentives. *bioarxiv*. https://doi.org/10.1101/2022.10.04.510790

Murphy, C., Dehmelt, V., Yonelinas, A. P., Ranganath, C., & Gruber, M. J. (2021). Temporal proximity to the elicitation of curiosity is key for enhancing memory for incidental information. *Learning & Memory*, *28*(2), 34-39. https://doi.org/10.1101/lm.052241.120
