# Supplementary material for: Autonomy in learning: Predictability modulates the beneficial effect of choice on memory
Source: Mem Cognit. 2026 Feb 25;54(6):2197–211. doi: 10.3758/s13421-026-01863-9 (PMC13461742; doi:10.3758/s13421-026-01863-9)
Supplement: Supplementary file 2 [file 13421_2026_1863_MOESM2_ESM.docx]

**Supplement 2: Secondary analyses**

For both experiments, we conducted additional analyses to get a better understanding of why the choice-related memory benefits are less strong for low predictability compared with high predictability. Specifically, we looked at (1) whether the choice effect in low predictability conditions was still diminished after omitting the not selected objects from the analyses and (2) whether the choice effect on memory accuracy differed between selected and not selected objects under low predictability.

**Inconsistency between selection and perceived information attenuates the choice effect on memory**

It should be noted that, in low predictability conditions, participants only saw the object picture corresponding to the selected object name in 50% of all trials. In the other 50% of the trials, participants were presented with the object picture that was not selected. In the latter case, there was inconsistency between the selection they made (selected object name) and the visual information (object picture) they perceived. On the contrary, in the high predictability conditions, all objects that participants saw were selected. Therefore, there is a possibility that the reduction of choice effect on memory accuracy under low predictability merely resulted from the inconsistency in the not selected trials. Two secondary analyses were implemented to investigate this assumption.

First, we conducted the same analysis as described for the primary analysis on selected trials only, with memory accuracy as the dependent variable. These are all trials from the high predictability conditions and half of the trials (trials in which the selected object names were consistent with the presented objects) from the low predictability conditions. The model included the main effects of “choice (yes/no)” and “predictability (high/low)”, as well as the interaction effect between “choice (yes/no)” and “predictability (high/low)” as fixed effects, as well as a full random effects structure per participant (Barr, 2013; Barr et al., 2013). If the inconsistency between the selected name and object picture could explain part of the interaction effect between choice and predictability in the primary analysis, then omitting the not selected trials would lead to insignificance of this interaction effect. Otherwise, the results should remain the same as the primary analysis before.

Second, we compared the choice effect for selected and not selected objects under low predictability to validate whether the choice effect only existed for selected objects. The model included the main effects of “choice (yes/no)” and “selection (yes/no)”, as well as the interaction effect between “choice (yes/no)” and “selection (yes/no)” as fixed effects, as well as a full random effects structure per participant (Barr, 2013; Barr et al., 2013). If the inconsistency between the selected name and object picture could diminish the choice effect, we would find that the choice effect for memory accuracy would not exist for not selected objects under low predictability.

**Results**

First, the same 2*2 analysis of choice and predictability effects was conducted as the primary analysis after omitting the not selected objects from low predictability conditions. In Experiment 1, we found the same main choice effect and interaction effect between choice and predictability (Fig. S2A, Table S3) as the primary analysis including both selected and not selected objects. However, in Experiment 2, the main choice effect was still significant, but the interaction effect was not significant anymore after omitting the not selected objects (Fig. S2C, Table S3). In other words, in Experiment 2, with the selected objects only, participants showed the same choice effect between high and low predictability.

Second, we separated the objects under low predictability into selected and not selected objects. We conducted a 2*2 analysis between choice conditions (choice or no-choice) and selection conditions (selected or not selected). In Experiment 1, we found the main effects of choice and selection (Fig. S2B, Table S3). This suggested that the decline of choice effect under low predictability in Experiment 1 happened for both selected and not selected objects.

However, in Experiment 2, we found the main effect of choice and the interaction between choice and selection. This suggested that after controlling object name viewing time in Experiment 2, the choice effect got smaller only when they saw a not selected object under low predictability (Fig. S2D, Table S3). It is also worth mentioning that the choice facilitatory effect remained significant for not selected objects.

**Summary**

In summary, these results indicated that inconsistency between the selected object name and object picture could explain part of the reduction of choice effect under low predictability. However, even if participants did not see the object they selected, they still remembered objects better when they could choose than when they could not choose. By conducting these secondary analyses, we found that the choice effect on memory under low predictability declined for both selected and not selected objects in Experiment 1. In contrast, the choice effect on memory only declined for not selected objects under low predictability in Experiment 2.

In Experiment 1, when people could control how much time they spent viewing the object names, under the low predictability condition, both selected and not selected objects had a smaller choice effect than under the high predictability condition. However, when the object name viewing time could not be controlled by the participants (Experiment 2), the choice effect for selected objects under low predictability remained the same as high predictability. The choice effect on memory did not reduce for the selected objects under low predictability, but it was diminished for not selected objects. It is worth noting that for both experiments, choice always notably enhanced memory accuracy for objects under high predictability, selected and not selected objects under low predictability.

Table S3 Linear Mixed Effect Model from Secondary Analyses

|  | **Experiment 1** | **Experiment 2** |
| --- | --- | --- |
| **Accuracy: primary analysis after omitting not selected objects** | | |
| Choice | *β* = 0.31, *z* = 1.94, *p* < 0.001 | *β* = 0.43, *z* = 7.69, *p* < 0.001 |
| Predictability | *β* = 0.19, *z* = 1.29, *p* = 0.82 | *β* = 0.05, *z* = 0.92, *p* = 0.34 |
| Choice * Predictability | *β* = -0.41, *z* = -1.96, *p* = 0.05 | *β* = -0.02, *z* = -0.41, *p* = 0.68 |
| *Follow-up t-tests:* |  |  |
| *Choice – No choice (High Pred)* | *β* = 0.72, *z* = 5.36, *p* < 0.001 | *β* = 0.89, *z* = 6.94, *p* < 0.001 |
| *Choice – No choice (Low Pred)* | *β* = 0.31, *z* = 1.94, *p* = 0.05 | *β* = 0.81, *z* = 4.97, *p* < 0.001 |
| *High Pred - Low Pred (Choice)* | *β* = 0.22, *z* = 1.54, *p* = 0.12 | *β* = 0.14, *z* = 1.00, *p* = 0.32 |
| *High Pred - Low Pred (No Choice)* | *β* = -0.19, *z* = -1.29, *p* = 0.20 | *β* = 0.06, *z* = 0.40, *p* = 0.69 |
| **Accuracy: comparing selected and not selected objects for low predictability** | | |
| Choice | *β* = 0.27, *z* = 1.83, *p* = 0.02 | *β* = 0.27, *z* = 4.16, *p* < 0.001 |
| Selection | *β* = 0.20, *z* = 1.59, *p* = 0.01 | *β* = 0.09, *z* = 1.70, *p* = 0.22 |
| Choice * Selection | *β* = -0.05, *z* = -0.31, *p* = 0.76 | *β* = -0.12, *z* = -2.49, *p* = 0.013 |
| *Follow-up t-tests:* |  |  |
| *Choice – No choice (Selected)* | *β* = 0.33, *z* = 2.00, *p* = 0.05 | *β* = 0.79, *z* = 4.59, *p* < 0.001 |
| *Choice – No choice (Not selected)* | *β* = 0.27, *z* = 1.83, *p* = 0.07 | *β* = 0.31, *z* = 2.05, *p* = 0.04 |
| *Selected - Not selected (Choice)* | *β* = 0.26, *z* = 1.98, *p* = 0.05 | *β* = 0.41, *z* = 2.69, *p* = 0.01 |
| *Selected - Not selected (No Choice)* | *β* = 0.20, *z* = 1.59, *p* = 0.11 | *β* = -0.06, *z* = -0.50, *p* = 0.62 |

Note:

Omitting not selected objects:

Memory accuracy ~ choice * predictability + (1 + choice * predictability|subject)

Selected or not selected objects for low predictability conditions:

Memory accuracy ~ choice * selection + (1 + choice * selection|subject)

Table S4Descriptive statistics for memory accuracy based on secondary analysis

|  |  | **Experiment 1 M ± SD** | **Experiment 2 M ± SD** |
| --- | --- | --- | --- |
| **Accuracy: omitting not selected objects** | | | |
| Choice main effect | Choice | 85.7% ± 11.9% | 85.3% ± 13.3% |
|  | No choice | 76.9% ± 16.0% | 75.0% ± 16.0% |
| Predictability main effect | High pred | 81.4% ± 13.4%) | 80.8% ± 14.1% |
|  | Low pred | 81.6% ± 13.8% | 79.1% ± 15.9% |
| Follow-up comparison | *Choice (High pred)* | *86.3% ± 12.6%* | *85.8% ± 13.3%* |
|  | *Choice (Low pred)* | *84.1% ± 13.8%* | *83.6% ± 16.2%* |
|  | *No choice (High pred)* | *75.6% ± 17.5%* | *75.4% ± 16.9%* |
|  | *No choice (Low pred)* | *78.8% ± 17.4%* | *73.6% ± 20.5%* |
| **Accuracy: selected or not selected objects for low predictability conditions** | | | |
| Choice main effect | Choice | 81.9% ± 14.9% | 81.1% ± 16.7% |
|  | No choice | 77.2% ± 17.3% | 73.6% ± 20.1% |
| selection main effect | Selected | 81.6% ± 13.8% | 79.1% ±15.9% |
|  | Not selected | 77.6% ± 16.9% | 76.2% ± 19.2% |
| *Follow-up comparison* | *Choice (Selected)* | *84.1% ± 13.8%* | *83.6% ± 16.2%* |
|  | *Choice (Not selected)* | *79.4% ± 17.7%* | *78.4% ± 19.5%* |
|  | *No choice (Selected)* | *78.8% ± 17.4%* | *73.6% ± 20.5%* |
|  | *No choice (Not selected)* | *75.3% ± 19.2%* | *73.4% ± 22.0%* |

*Figure S2. Secondary analyses results.*

***A & B, Experiment 1.*** *A. In this panel, we only included the trials in which objects were selected and seen, and the same effects as the primary analysis in Figure 2A were found, namely the main effect of choice and the interaction effect between choice and predictability on memory accuracy. This indicated that the choice effect was larger under high predictability than under low predictability. B. Only objects in the low predictability condition were considered, and they were categorized as either selected or not selected. We compared the choice effect on memory accuracy for selected and not selected objects. It was found that in the low predictability conditions, the selected or not selected objects showed similar choice effects. Taken together, in Experiment 1, the reason for the choice effect reduction under low predictability was that the choice effect was smaller for both selected and not selected objects.*

***C & D, Experiment 2.*** *C. In this panel, akin to Panel A (Experiment 1), we exclusively considered the objects that were selected and conducted the same analysis. In contrast to Experiment 1, we only found a main effect of choice on memory accuracy. This indicated that the choice effect on memory accuracy remained the same between the high and low predictability after omitting objects that were not selected. D. In this panel, we analyzed the choice effect for selected or not selected objects under the low predictability conditions, the same as Panel B (Experiment 1). We found that the choice effect and interaction effect between choice and selection conditions were significant. In other words, when object name viewing time was controlled, choice had a more pronounced impact on selected objects compared to non-selected objects, which differs from the findings of Experiment 1. It is worth mentioning that the choice effect remained significantly positive even for the not selected objects. To summarize, in Experiment 2, the decline of the choice effect from high to low predictability (Figure 2C, the slope for the red line was bigger than the blue line) was caused mainly by the not selected objects.*

**References**

Barr, D. J. (2013). Random effects structure for testing interactions in linear mixed-effects models. *Frontiers in Psychology*, *4*, 328. https://doi.org/10.3389/fpsyg.2013.00328

Barr, D. J., Levy, R., Scheepers, C., & Tily, H. J. (2013). Random effects structure for confirmatory hypothesis testing: Keep it maximal. *Journal of Memory and Language*, *68*(3). https://doi.org/10.1016/j.jml.2012.11.001
