# Supplementary material for: Autonomy in learning: Predictability modulates the beneficial effect of choice on memory
Source: Mem Cognit. 2026 Feb 25;54(6):2197–211. doi: 10.3758/s13421-026-01863-9 (PMC13461742; doi:10.3758/s13421-026-01863-9)
Supplement: Supplementary file 3 [file 13421_2026_1863_MOESM3_ESM.docx]

**Supplement 3: object name viewing time**

In Experiment 1, we found that participants remembered objects better in choice than in no-choice conditions. Meanwhile, this choice effect on memory accuracy was attenuated when participants could not predict the outcome of their choices well. However, in Experiment 1, the object names would disappear once participants pressed a button (Fig. 1A). Hence, participants could control not only the content (which picture they would like to see) but also the object name viewing time (the amount of time that each pair of words was presented on the screen). In the current setup, object names potentially functioned as cues for the pictures that participants needed to remember, which could facilitate memory encoding and formation (Crouse & Idstein, 1972; Neumann & Strack, 2000; Thomson & Tulving, 1970). In other words, the longer participants could view the object names, the better they would remember the objects that the names are attributed.

To summarize, if we find similar effects of choice and predictability on object name viewing time as for memory accuracy, it might be the case that (part of) the findings of memory accuracy can be explained by the object name (cue) viewing time differences.

**Methods**

For this analysis, we used the object name viewing time in Experiment 1 as a dependent variable. We conducted the same analysis as the primary analysis (2 * 2 LME model including choice and predictability main effect and the interaction effect) described in the main text (Barr, 2013; Barr et al., 2013). In accordance with the primary analysis on memory accuracy, this analysis on object name viewing time also only included confident trials. Before conducting inferential statistical analysis on object name viewing time, we log transferred the raw viewing time to gain a normally distributed dependent variable.

**Results and conclusion**

Similar to the results on memory accuracy (Fig. 2A), we found the main effect of choice and the interaction effect between choice and predictability on object name viewing time (Table S6, Fig. S3). Therefore, according to these results, we designed Experiment 2 to control the object name viewing time to control the strength of the cues, isolate the influence of the cues, and validate our findings.

Table S5 Linear mixed effect model for object name viewing time, Experiment 1

| **Object name viewing time: confident trials** |  |
| --- | --- |
| Choice | *β* = 0.43, *t* = 9.90, *p* < 0.001 |
| Predictability | *β* = 0.02, *t* = 0.75, *p* = 0.08 |
| Choice * Predictability | *β* = 0.13, *t* = 3.14, *p* = 0.003 |
| *Follow-up t-tests:* |  |
| *Choice – No choice (High Pred)* | *β* = 0.56, *t* = 12.00, *p* < 0.001 |
| *Choice – No choice (Low Pred)* | *β* = 0.43, *t* = 9.90, *p* < 0.001 |
| *High Pred - Low Pred (Choice)* | *β* = 0.15, *t* = 3.58, *p* < 0.001 |
| *High Pred - Low Pred (No Choice)* | *β* = 0.02, *t* = 0.75, *p* = 0.46 |

Note: Object name viewing time ~ choice * predictability + (1 + choice * predictability|subject)

Table S6 Descriptive statistics for object name viewing time, Experiment 1

| **Object name viewing time: confident trials (M ± SD)** | | |
| --- | --- | --- |
| Choice main effect | Choice | 1211ms ± 442ms |
|  | No choice | 717ms ± 244ms |
| Predictability main effect | High pred | 1020ms ± 353ms |
|  | Low pred | 927ms ± 318ms |
| Follow-up comparison | *Choice (High pred)* | *1295ms ± 510ms* |
|  | *Choice (Low pred)* | *1122ms ± 428ms* |
|  | *No choice (High pred)* | *717ms ± 251ms* |
|  | *No choice (Low pred)* | *717ms ± 264ms* |

*Figure S3. The same analysis as the primary analysis (Fig. 2A) was conducted but with object name viewing time as the dependent variable.There was a main effect of choice and no main effect of predictability on object name viewing time. Also, the interaction between choice and predictability on object name viewing time was found. These results were similar to the results found on memory accuracy depicted in the main text (Fig.2). These suggested that participants would view the object names longer when they have a choice than no choice, and the choice effect is bigger under high predictability than low predictability. In the current paradigm, object names can be seen as cues facilitating memory of the pictures. Since longer exposure to cues might result in better memory (e.g., Crouse & Idstein, 1972), the object name viewing time should be controlled. We designed Experiment 2 based on this finding.*

**References**

Barr, D. J. (2013). Random effects structure for testing interactions in linear mixed-effects models. *Frontiers in Psychology*, *4*, 328. https://doi.org/10.3389/fpsyg.2013.00328

Barr, D. J., Levy, R., Scheepers, C., & Tily, H. J. (2013). Random effects structure for confirmatory hypothesis testing: Keep it maximal. *Journal of Memory and Language*, *68*(3). https://doi.org/10.1016/j.jml.2012.11.001

Crouse, J. H., & Idstein, P. (1972). Effects of encoding cues on prose learning. *Journal of Educational Psychology*, *63*(4), 309-313.

Neumann, R., & Strack, F. (2000). Approach and avoidance: The influence of proprioceptive and exteroceptive cues on encoding of affective information. *Journal of Personality and Social Psychology*, *79*(1), 39-48.

Thomson, D. M., & Tulving, E. (1970). Associative encoding and retrieval weak and strong cues. *Journal of Experimental Psychology*, *86*(2), 255-262.
